# Memory-Guided Saccades and Non-Motor Symptoms Improve after Botulinum Toxin Therapy in Cervical Dystonia

**DOI:** 10.3390/jcm13195708

**Published:** 2024-09-25

**Authors:** Tihana Gilman Kuric, Zvonimir Popovic, Sara Matosa, Aleksander Sadikov, Vida Groznik, Dejan Georgiev, Alessia Gerbasi, Jagoda Kragujevic, Tea Mirosevic Zubonja, Zdravka Krivdic Dupan, Silva Guljas, Igor Kuric, Stjepan Juric, Ruzica Palic Kramaric, Svetlana Tomic

**Affiliations:** 1 Department of Neurology, Osijek University Hospital Center, 31000 Osijek, Croatia; zvonimir.popovic@kbco.hr (Z.P.); sara.matosa@kbco.hr (S.M.); jagoda.kragujevic@kbco.hr (J.K.); tea.mirosevic@hlk.hr (T.M.Z.); sjuric@mefos.hr (S.J.); ruzica.palic-kramaric@kbco.hr (R.P.K.); svetlana.tomic@kbco.hr (S.T.); 2 Faculty of Medicine in Osijek, Josip Juraj Strossmayer University of Osijek, 31000 Osijek, Croatia; zdravka.krivdic@gmail.com (Z.K.D.); silva.guljas@kbco.hr (S.G.); igorkuric13@gmail.com (I.K.); 3 Faculty of Computer and Information Science, University of Ljubljana, 1000 Ljubljana, Slovenia; aleksander.sadikov@fri.uni-lj.si (A.S.);; 4 Department of Neurology, Ljubljana University Medical Centre, 1000 Ljubljana, Slovenia; dejan.georgiev@kclj.si; 5 Department of Electrical, Computer and Biomedical Engineering, University of Pavia, 27100 Pavia, Italy; alessia.gerbasi@unipv.it; 6 Faculty of Dental Medicine and Health, Josip Juraj Strossmayer University of Osijek, 31000 Osijek, Croatia; 7 Department of Diagnostic and Interventional Radiology, Osijek University Hospital Center, 31000 Osijek, Croatia

**Keywords:** cervical dystonia (CD), botulinum toxin (BoNT/A), central effect, non-motor symptoms, eye tracker

## Abstract

**Background/Objectives:** Cervical dystonia (CD) is a condition characterized by involuntary activity of cervical muscles, which is often accompanied by various non-motor symptoms. Recent studies indicate impaired saccadic eye movements in CD. Local administration of botulinum toxin type A (BoNT/A), which causes temporary paralysis of the injected muscle, is the first-line treatment of focal dystonia, including CD. To our knowledge, concurrent observation of the effect of BoNT/A on smooth eye movements, voluntary saccades, memory-guided saccades, and antisaccades in CD has not yet been explored. The aim of this study was to assess the effect of BoNT/A on eye movements and non-motor symptoms in patients with CD, which, when altered, could imply a central effect of BoNT/A. **Methods:** Thirty patients with CD performed smooth pursuit, prosaccadic expression, memory-guided saccades, and antisaccade tasks; eye movements were recorded by an eye tracker. Motor and non-motor symptoms, including depression, anxiety, pain, disability, and cognitive changes prior to and after BoNT/A administration, were also evaluated. **Results:** The number of correct onward counts (*p* < 0.001), overall correct memory-guided saccades count (*p* = 0.005), motor symptoms (*p* = 0.001), and non-motor symptoms, i.e., anxiety (*p* = 0.04), depression (*p* = 0.02), and cognition (*p* < 0.001) markedly improved after BoNT/A administration. **Conclusions:** Memory-guided saccades, depression, and anxiety improve after BoNT/A in CD.

## 1. Introduction

Cervical dystonia (CD) is a condition characterized by involuntary activity of the cervical muscles leading to abnormal postures of the head, neck, and shoulders, and these postures are often worsened by voluntary action due to excessive muscle activation [1]. In addition to motor symptoms, CD includes non-motor symptoms such as anxiety, depression, cognitive problems, pain, sexual dysfunction, and sleeping disorders, which occur in about 36% of patients [2]. The gold standard in the treatment of CD is botulinum toxin type A (BoNT/A) [3,4]. The latest studies do not consider CD to be a disorder of a certain brain structure but rather a network disorder involving the so-called neural integrator, a neural network formed by connections between the basal ganglia, the cerebellum, and proprioceptive feedback that ensures the normal position of the head when processing external stimuli and planning and executing movements [5]. The pathopsysiology of non-motor symptoms has been linked to the corticobasal circuits involving the frontal cortex, who also control executive functions, saccadic eye movements, motor activity, motivation, and certain aspects of behavior [6,7]. The neural pathways responsible for eye movements have long been used in numerous studies as a model for research into motor control, as they require elements of planning, synchronization, and execution that take place using higher brain functions. Oculomotor function in CD at bedside investigation usually appears normal and, therefore, has not been the main interest of much scientific research, which so far shows inconsistent results, as some studies have reported asymmetric vestibuloocular reflexes and impaired inhibitory control of saccadic eye movements [8,9,10]. In contrast, other studies observed no difference compared with healthy controls [11]. Given that the brain pathway for guiding visual saccades extends from the cerebral cortex to the brainstem and includes the corticobasal circuits that connect the frontal region, basal ganglia, and the cerebellum [12,13], new research has focused on the examination of eye movements in CD assuming, among other things, the existence of pathology within the neural integrators of eye motility [14].

To the best of our knowledge, so far, no study has explored the effect of BoNT/A on smooth pursuit, prosaccadic expression, antisaccades, and memory-guided saccades simultaneously in CD. The aim of this study was to assess the effect of BoNT/A on eye movements and non-motor symptoms in patients with CD, which, when altered, could imply a central effect of BoNT/A.

## 2. Materials and Methods

### 2.1. Participants and Inclusion and Exclusion Criteria

Our single-center study gathered 30 participants with idiopathic CD from the Department of Neurology, University Hospital Center Osijek, Croatia. The exclusion criteria were a history of brain damage of any etiology and recently diagnosed dementia, use of drugs that could affect cognitive functions or induce iatrogenic movement disorders, and visual impairments that could affect eye movements (severe myopia, astigmatism, strabismus, low vision, and cataracts). Mild and moderate myopia or presbyopia were not considered exclusion criteria if they were adequately corrected. The study was approved by the Ethics Committee of Osijek University Hospital Center (study number R2-6782/2018). Signed informed consent was obtained from all participants. 

### 2.2. Eye Movement Evaluation

Four types of eye movements were simultaneously analyzed (smooth eye movements, voluntary saccades, memory-guided saccades, and antisaccades) using a Tobii TX300 eye tracker (https://www.spectratech.gr/Web/Tobii/pdf/TX300.pdf, accessed on 25 September 2024). The software used in the research was developed in the Laboratory for Artificial Intelligence at the Faculty of Computer and Information Science of the University of Ljubljana. Before each task, the patient was given clear written and textual instructions on the screen as to how the task should be performed, and a short test/calibration of each task was performed to confirm that the patient understood the task correctly. Smooth eye movements were tested by tracking a white dot (target) moving along the horizontal (x) and vertical (y) axes. Voluntary saccades were examined so that the subject had to focus their gaze on the target (white dot) in the middle of the screen; the target disappeared and, after a few seconds, appeared on the left or right half of the screen, whereby the subject was asked at the beginning of the test to direct their gaze towards the target. When testing antisaccades, subjects were asked to fix their gaze on the target in the middle of the screen, but this time in the direction opposite to the appearance of the target once it disappeared from the screen and appeared in the left or right half of the visual field. Testing of memory saccades was carried out so that the patient had to follow the squares that lit up in a certain order on the screen, whereby the task was to repeat the exact sequence of lighting the squares in the order in which they appeared. In doing so, two testing methods were performed, as the lighting of squares was memorized in the normal order (forward) and also backward (in which the patient had to fixate on the last square up to the first lit square). Memory saccade testing consisted of both test version A (used before BoNT/A) and test version B (used after BoNT/A) to avoid repetition and to prevent the participants from learning correct answers. The visual test parameters were tested as follows: for voluntary saccades, one cycle of the performed movement during 1600 ms was evaluated, and prosaccade evaluated the mean square error during pursuit delay/latency, general speed, and accuracy. Antisaccades were monitored to evaluate the antisaccade reaction time (latency), antisaccade directional errors, and countermanding inhibition errors in milliseconds, while memory saccades were monitored to evaluate the number of correct onward counts, correct backward counts, and overall correct counts. Before each examination, a test examination consisting of identical tasks was carried out so that the subjects and the examiner could ensure that the task was properly understood. There was a 30-s break between each task. The software contained A and B versions composed of different memory-saccade and antisaccade tasks for randomization purposes.

### 2.3. Motor and Non-Motor Symptom Evaluation; BoNT/A Administration

Motor symptoms were evaluated using the Tsui score [15]. Participants filled in the Toronto Western Spasmodic Torticollis Rating Scale (TWSTRS) questionnaire [16] to evaluate pain and disability. The Beck Anxiety Inventory (BAI) and Beck Depression Inventory (BDI-II) were used for the evaluation of psychiatric symptoms [17,18]. Addenbrooke’s Cognitive Examination Rating scale (ACE-R) was used to evaluate cognitive functions [19]. Motor symptoms, evaluation of pain and disability, psychiatric symptoms, and cognitive status were examined on two occasions, i.e., before and after BoNT/A treatment. Participants were allowed to enter the study if they were BoNT/A-free for at least 3 months before the initial testing and drug administration. Eye movement testing was performed 4 weeks after BoNT/A administration in order to ensure sufficient time for BoNT/A action. The BoNT/A administration scheme was individually selected for each patient based on their symptoms, according to the COL-CAP concept. The manufacturer of INCO used in this research was Merz Pharma GmbH & Co. KgaA, Frankfurt/Main, Germany. The toxin came in the form of a powder solution that was suitable for injection and was reconstituted prior to use with sodium chloride 9 mg/mL (0.9%) solution (1 cc into a 100 U of BoNT/a vial or 0.5 cc in 50 U of BoNT/A vial). A 20–27 G short-bevel sterile needle was used for reconstitution. After the solvent was drawn up into a syringe, it was injected into the vial, mixed with INCO powder, and gently stirred to form a clear, colorless solution. Each patient received between 100 and 250 units of incoBoNT/A. All tests were performed by one person under the same conditions (in a well-lit, quiet room) in the morning hours.

The examination of eye movements was always performed first and lasted for 40 min on average. This was followed by a short break, after which all other motor and non-motor tests (TSUI, TWSTRS, BAI, BDI-II, ACE-R) were performed. The overall duration of the examination was 90–120 min on average. 

### 2.4. Data Analysis

The Wilcoxon signed-rank test was used to test for differences in the measured variables before and after BoNT/A administration. The data were statistically analyzed using SPSS v24 [20]. The level of significance for all analyses was set at a 2-sided *p* < 0.05.

## 3. Results

Motor and non-motor symptoms and characteristics of eye movements were studied in 30 patients (21 females and 9 males) with CD. The mean age, duration of CD, duration, and dosage of BoNT/A are presented in Table 1. 

### 3.1. Motor Symptoms in Patients with CD before and after BoNT/A

After BoNT/A treatment, a significant improvement in motor symptoms (total Tsui score) (*p* = 0.001) was observed (Table 2) in terms of reduced involuntary rotation, tilting, and head/neck deflection (Tsui A) (*p* = 0.003), while there were no significant differences in the strength and duration of involuntary movements (Tsui B), shoulder elevation (Tsui C), or the strength and duration of tremors (Tsui D).

### 3.2. Non-Motor Symptoms in Patients with CD before and after BoNT/A

After BoNT/A administration, there was a significant improvement in most of the non-motor symptoms (cognition, pain, disability, anxiety, and depression) measured (Table 3). Concerning psychiatric symptoms, a reduction in anxiety (*p* = 0.04) and depression (*p* = 0.02) was recorded, followed by a significant reduction in disability (*p* = 0.004) and pain subscales (*p* = 0.001) of the TWSTRS. The improvement in overall cognitive abilities (*p* < 0.001) included improved memory (*p* = 0.003), verbal fluency (*p* = 0.002), and language abilities (*p* = 0.02). There were no improvements in visuospatial skills or attention.

### 3.3. Eye Movements in Patients with CD before and after BoNT/A

The measured variables of smooth eye movements before and after botulinum toxin therapy did not show significant improvements in smooth movements, voluntary saccades, or antisaccades (Table 4). There was no significant difference in smooth pursuit or in prosaccadic expression, with the latter showing a slight improvement regarding latency, speed, and accuracy compared with the mean values before and after therapy; however, without a significant difference. Although there was no significant difference in the length and the number of correct backward counts, the number of correct onward counts (*p* < 0.001) and number of overall memory-guided saccade sequence correct counts (*p* = 0.005) improved significantly after botulinum toxin therapy (Table 4). 

## 4. Discussion

The main objective of the study was to examine the effect of BoNT/A on eye movement and non-motor symptoms, which, when altered, could imply central effects of BoNT/A. Memory-guided saccade sequences—more specifically, the correct onward and overall correct count—improved significantly after BoNT/A treatment. There was no significant improvement in smooth pursuit, prosaccadic expression, or antisaccades in any gaze direction. BoNT/A also showed a tendency towards a slight improvement in the correct antisaccade reaction time (*p* = 0.06), directional errors (*p* = 0.06), and countermanding inhibition errors (*p* = 0.07), which are standard methods for assessing cognitive/inhibitory control over action in eye movements.

Memory-guided saccades rely on information stored in the memory to guide the eyes to a remembered location when there is no visual stimulus, depending mostly on the frontal eye field (FEF) and the supplementary eye field (SEF) to control more complex sequences of saccades or saccades in combination with body movements [21]. Antisaccades are under voluntary control and demand, suppressing the reflex saccadic response to the presented target. At least two inhibition mechanisms are required to perform antisaccade movements, relying on intact FEFs and superior colliculus to avoid expressing errors in antisaccade reaction time [21]. In contrast, when a stimulus appears, automatic saccades targeting is suppressed by the SEFs. Disruption of this link results in a longer antisaccade reaction time [21,22]. The improvement in memory-guided saccades but lack of a significantly better performance in antisaccade tasks after the administration of BoNT/A in our study (considering the involvement of both FEFs and SEFs in each case) can be explained by the independence of memory-guided saccade neural systems from the ones that generate visually presented antisaccades [23,24]. This is consistent with the idea that movement control may be guided by perceptual memory without a presented target [25,26], pointing to a difference in the spatial representation of the target used to program visually guided saccades as opposed to that used to program memory-guided saccades [27]. Another reason for the observed changes in memory-guided saccades, but not of other eye movements, could be explained by the proposed theory of parallel organization of functionally separate network circuits connecting the basal ganglia and the cortex [28], assuming that impulses from the basal ganglia through separate network circuits stimulate different parts of the frontal lobe, although the striatum receives impulses from the entire neocortex [29]. 

Our results also report significant changes in non-motor symptoms after therapy administration (Table 3), showing a significantly reduced degree of anxiety and depression on the BAI and BDI-II test assessments, as well as an evident pain reduction. Also, a comparison of the ACE-R test of cognitive abilities after BoNT/A administration in our study shows a significant general improvement in cognitive abilities in the form of memory, speech fluency, and speech (Table 3). Cognitive impairment in patients with CD is still a controversial topic; some studies have shown improvements in attention [30] after the administration of BoNT/A in CD patients; according to others, there are notable differences in attention and executive functioning [31] in CD patients compared to healthy controls; while in a comparison of the cognitive status of the genetically inherited type of dystonia DYT1, no difference was found compared to healthy controls [29].

What could explain the improvement in memory-guided saccades and non-motor symptoms of CD patients after BoNT/A therapy?

The reduction in pain was previously attributed to the peripheral effect of botulinum toxin, which results in a reduction in muscle spasms, but some studies have shown that as in some patients with Parkinson’s disease [28], the level of pain in some patients with CD does not correlate with the severity of motor symptoms. It was observed that patients with a similar clinical presentation report different pain intensities [32,33], while some show a significant reduction in pain with no significant improvement in motor symptoms [34]. The mentioned works and the conducted research imply that the pain that occurs in patients with CD is partly of central origin. Numerous works have, for some time, considered psychiatric symptoms, cognitive disturbances, and pain as separate entities within CD that are not related to motor symptoms [35,36]. Constanzo et al. [34] demonstrated that BoNT-A intramuscular injection was able to improve non-motor variables (psychiatric symptoms and pain) in patients affected by CD. A prospective observational study conducted by Sugar D. et al. [37] showed that BoNT leads to improvement in both motor severity and anxiety in CD. Similar to our results, an improvement in anxiety did not correlate well with improvements in motor severity during the time of known peak effect, demonstrating that this effect on anxiety is not secondary to the degree of improvement in motor symptom severity and could, as such, indicate a direct beneficial effect of BoNT on anxiety. This is supported by the work of Moriarty Al et al. [38], whose longitudinal follow-up study of mood in CD showed that anxiety and depressive symptoms persist in cervical dystonia, and these effects are seemingly unrelated to pain severity. Considering their possible influence on cognitive abilities, BoNTs are reported to affect inhibitory, excitatory, and sensory neurons as well as several genes related to proteasomal degradation pathways, neurite outgrowth, and inflammatory pathways [39], which could explain the observed improvement in overall cognitive abilities in our study. Nevertheless, it is still debatable whether cognitive changes among CD patients are the result of motor symptoms, pain, accompanying anxiety and depression, or a primary element within CD.

The majority of basic and clinical research has been largely focused on the peripheral effects of BoNTs, but most evidence from animal models and human studies has also confirmed BoNTs’ actions at the central nervous system level [40]. The proven central effect may be the consequence of hematogenous spread, retrograde neural transport of BoNTs to the central nervous system (CNS), or indirect action due to denervation and changes in afferent input resulting in plastic reorganization of the CNS via changes to sensory afferents [41,42]. There is evidence of direct retrograde axonal transport of binding fragments of BoNT/A with a speed of retrograde transport of 0.8 μm/sec, including long-distance transport of BoNT/A from the hind leg muscles of adult rats confirmed by detection of cl-SNAP25 (BoNT/A target) in spinal cord motor neurons [43] or by demonstrating significant amounts of BoNT/A-truncated SNAP-25 in the facial nucleus of rats via Western blot analysis after BoNT/A injection into the whisker pad [44]. Since experiments on rats are not exact concerning retrograde axonal transport in humans, human studies were conducted [45,46], supporting the idea of a direct central effect of muscular injected BoNT/A by reduction in spinal inhibition. Support for the indirect central effects of BoNTs originates from the observation that not all clinical effects of peripheral I.M. injections can be explained by the exclusive action of BoNTs on peripheral nerve terminals, suggesting a change in the functional organization of the CNS through an alteration mechanism induced by altered peripheral inputs [35]. The most significant neuroimaging evidence for the central effects of BoNT comes from functional magnetic resonance imaging (fMRI) studies. Opavsky et al. [47] demonstrated a reduced extent of hand movement-related cortical activation in CD patients 4 weeks after BoNT/A treatment, together with extensive changes in the contralateral secondary somatosensory cortex and altered activation of the ipsilateral supplementary motor area and dorsal premotor cortex. Using resting-state fMRI, Delnooz et al. [48] demonstrated both increased and decreased connectivity in sensorimotor and executive control networks in CD patients several weeks after BoNT/A therapy, whereas Brodoehl et al. [49] reported increased connectivity between the basal ganglia and sensorimotor network, together with the loss of functions in the putamen, thalamus, and somatosensory cortex. Altered sensorimotor integration is considered to be a significant part of CD pathophysiology, as previously stated; Nevrlý M. et al. demonstrated decreased functional connectivity within the somatosensory cortex [50], more specifically within the putamen, sensorimotor cortex, thalamus, and subthalamic nucleus, where it is speculated that by weakening dystonic muscles, BoNT/A creates sensory–motor discrepancy and influences the motor output of the brain by reducing effective movement through BoNT application so that the load of sensorimotor integration is globally reduced [49]. 

In addition, we could reliably show a difference in the correct onward count and overall memory-guided saccade correct count before and after botulinum toxin injections in CD. There were trends towards improvement of antisaccade directional errors and countermanding inhibition errors that could have reached statistical significance with a higher number of subjects. Therefore, future studies should include more participants.

## 5. Conclusions

Considering the marked improvement in memory-guided saccades (with no other significant changes in ocular movements) and improvement in non-motor symptoms (anxiety, depression, and cognition) after local BoNT/A therapy, our results suggest a possible indirect central effect of BoNT, although a peripheral neuromodulatory effect could not be excluded either. Further studies are needed to explore the effect of BoNT/A on eye movements and non-motor symptoms in CD.

## Figures and Tables

**Table 1:** Demographic data on age, duration of cervical dystonia, duration of BoNT/A treatment, and dosage of BoNT/A.

Demographic Data	Median (IQR)
Age (years)	63.00 (53.00–69.25)
CD duration (years)	10.00 (8.75–13.00)
BoNT/A therapy median (years)	7.50 (4.75–9.00)
BoNT/A dosage (units)	150.00 (150.00–250.00)

CD—cervical dystonia; BoNT/A—botulinum toxin type A; IQR—interquartile range.

**Table 2:** Assessment of motor symptoms before and after BoNT/A.

Tsui Scale	Median (IQR)		*p*
	Before BoNT/A	After BoNT/A	
Tsui A (amplitude of sustained movements)	2.5 (2.0–4.0)	2.0 (1.0–2.25)	0.003
Tsui B (duration of sustained movements)	2.0 (1.0–2.0)	2.0 (1.0–2.0)	0.058
Tsui C (shoulder elevation)	1.0 (0.0–2.0)	1.0 (0.0–1.0)	0.124
Tsui D (tremor)	1.0 (0.0–2.0)	1.0 (0.0–2.0)	0.206
Total score [(A) × (B)] + (C) + (D)	6.5 (4.75–9.25)	5.0 (3.0–6.25)	0.001

Wilcoxon test; IQR—interquartile range; BoNT/A—botulinum toxin type A.

**Table 3:** Assessment of non-motor symptoms (cognition, anxiety, depression, and pain) before and after BoNT/A.

Non-Motor Symptom Scale	Median (IQR)		*p*
	Before BoNT/A	After BoNT/A	
ACE-R Domains			
Orientation	10.0 (10.0–10.0)	10.0 (10.0–10.0)	>0.99
Attention	7.5 (6.0–8.0)	8.0 (6.0–8.0)	0.810
Memory	23.5 (17.0–25.0)	24.0 (22.25–26.0)	0.003
Verbal fluency	10.5 (7.0–12.0)	12.0 (10.0–14.0)	0.002
Language	25.0 (23.0–26.0)	26.0 (24.75–26.0)	0.02
Visuospatial skills	16.0 (16.0–16.0) (9–16) ^†^	16.0 (15.0–16.0) (13–16) ^†^	0.30
ACE-R total	89.0 (79.0–95.0)	94.5 (87.0–98.0)	<0.001
BAI	5.0 (1.0–20.0)	4.0 (2.0–15.0)	0.04
BDI-II	4.5 (2.0–8.0)	3.0 (1.0–5.0)	0.02
TWSTRS disability	14.5 (8.5–20.5)	6.0 (2.0–13.0)	0.004
TWSTRS pain	7.75 (4.69–9.75)	4.5 (0.0–8.0)	0.001

Wilcoxon test; ^†^ general range; IQR—interquartile range; CD—cervical dystonia; BoNT/A—botulinum toxin type A; BAI—Beck Anxiety Inventory; BDI-II—Beck Depression Inventory II; TWSTRS—Toronto Western Spasmodic Torticollis Rating Scale; ACE-R—Adenbrooke’s Cognitive Examination Rating Scale.

**Table 4:** Results of eye-tracking tasks before and after BoNT/A treatment.

Variable	Median (IQR)		*p*
	Before BoNT/A	After BoNT/A	
Smooth pursuit			
Horizontal prosaccade expression 1600 ms	0.5804 (0.5161–0.6503)	0.5619 (0.5226–0.6386)	0.48
Horizontal prosaccade reaction time 1600 ms	0.007779 (0.06382–0.01273)	0.007464 (0.0054950.01302)	0.66
Vertical prosaccade expression 1600 ms	0.5591 (0.4780–0.6344)	0.5327 (0.4978–0.5943)	0.60
Vertical prosaccade reaction time 1600 ms	0.01078 (0.006189–0.01437)	0.006783 (0.005085–0.01302)	0.42
Prosaccadic expression (MSE)			
Latency	7.59 (7.34–7.99)	7.37 (6.91–7.86)	0.57
Speed	2.86 (2.58–3.19)	2.99 (2.79–3.18)	0.77
Accuracy	3.74 (2.81–4.71)	3.21 (2.91–4.15)	0.52
Memory-guided saccade sequence			
Correct onward count	0.0 (0.0–4.0)	4.0 (0.0–6.0)	<0.001
Correct backward count	0.0 (0.0–1.0)	2.0 (0.0–5.0)	0.054
Overall correct count	0.0 (0.0–5.0)	5.5 (0.0–9.0)	0.005
Antisaccades	ms	ms	
Reaction time/latency	522.9 (479.5–538.1)	510.2 (443.8–605.4)	0.87
Antisaccade directional errors	0.0 (0.0–2.0)	1.0 (0.0–4.0)	0.06
Countermanding inhibition errors	2.0 (0.0–6.25)	1.0 (0.0–2.25)	0.07

Wilcoxon test; MSE—mean square error; IQR—interquartile range; CD—cervical dystonia; BoNT/A—botulinum toxin type A.

## Data Availability

The data presented in this study are available on request from the corresponding author.

## References

[1] Defazio G., Jankovic J., Giel J.L., Papapetropoulos S. (2013). Descriptive epidemiology of cervical dystonia. Tremor Other Hyperkinetic Mov..

[2] Ray S., Pal P.K., Yadav R. (2020). Non-Motor Symptoms in Cervical Dystonia: A Review. Ann. Indian Acad. Neurol..

[3] Patel S., Martino D. (2013). Cervical dystonia: From pathophysiology to pharmacotherapy. Behav. Neurol..

[4] Rasetti-Escargueil C., Palea S. (2024). Embracing the Versatility of Botulinum Neurotoxins in Conventional and New Therapeutic Applications. Toxins.

[5] Sedov A., Usova S., Semenova U., Gamaleya A., Tomskiy A., Crawford J.D., Corneil B., Jinnah H.A., Shaikh A.G. (2019). The role of pallidum in the neural integrator model of cervical dystonia. Neurobiol. Dis..

[6] Leisman G., Melillo R. (2013). The basal ganglia: Motor and cognitive relationships in a clinical neurobehavioral context. Rev. Neurosci..

[7] Stamelou M., Edwards M.J., Hallett M., Bhatia K.P. (2012). The non-motor syndrome of primary dystonia: Clinical and pathophysiological implications. Brain.

[8] Kassavetis P., Kaski D., Anderson T., Hallett M. (2022). Eye Movement Disorders in Movement Disorders. Mov. Disord. Clin. Pract..

[9] Carbone F., Ellmerer P., Ritter M., Spielberger S., Mahlknecht P., Hametner E., Hussl A., Hotter A., Granata R., Seppi K. (2021). Impaired Inhibitory Control of Saccadic Eye Movements in Cervical Dystonia: An Eye-Tracking Study. Mov. Disord. Off. J. Mov. Disord. Soc..

[10] Beck R.B., Kneafsey S.L., Narasimham S., O’Riordan S., Isa T., Hutchinson M., Reilly R.B. (2018). Reduced frequency of ipsilateral express saccades in cervical dystonia: Probing the nigro-tectal pathway. Tremor Other Hyperkin. Mov..

[11] Stell R., Bronstein A.M., Gresty M., Buckwell D., Marsden C.D. (1990). Saccadic function in spasmodic torticollis. J. Neurol. Neurosurg. Psychiatry.

[12] Sparks D.L. (1989). The neural encoding of the location of targets for saccadic eye movements. J. Exp. Biol..

[13] Robinson F.R., Fuchs A.F. (2001). The role of the cerebellum in voluntary eye movements. Annu. Rev. Neurosci..

[14] Shaikh A.G., Zee D.S., Crawford J.D., Jinnah H.A. (2016). Cervical dystonia: A neural integrator disorder. Brain.

[15] Tsui J.K., Eisen A., Stoessl A.J., Calne S., Calne D.B. (1986). Double-blind study of botulinum toxin in spasmodic torticollis. Lancet.

[16] Consky E., Basinki A., Belle L., Ranawaya R., Lang A. (1990). The Toronto Western Spasmodic Torticollis Rating Scale (TWSTRS): Assessment of validity and inter-rater reliability. Neurology.

[17] Beck A.T., Steer R.A. (1993). Manual for the Beck Anxiety Inventory.

[18] Wang Y., Gorenstein C. (2013). Assessment of depression in medical patients: A systematic review of the utility of the Beck Depression Inventory-II. Clinics.

[19] Bruno D., Schurmann Vignaga S. (2019). Addenbrooke’s cognitive examination III in the diagnosis of dementia: A critical review. Neuropsychiatr. Dis. Treat..

[20] BM Corp (2016). IBM SPSS Statistics for Windows, Version 24.0.

[21] Purves D., Augustine G.J., Fitzpatrick D., Katz L.C., LaMantia A.S., McNamara J.O., Williams S.M. (2001). Neural Control of Saccadic Eye Movements. Neuroscience.

[22] Mitchell J.F., Zipser D. (2003). Sequential memory-guided saccades and target selection: A neural model of the frontal eye fields. Vis. Res..

[23] Funahashi S., Bruce C.J., Goldman-Rakic P.S. (1989). Mnemonic coding of visual space in the monkey’s dorsolateral prefrontal cortex. J. Neurophysiol..

[24] Hikosaka O., Wurtz R.H. (1985). Modification of saccadic eye movements by GABA-related substances. I. Effect of muscimol and bicuculline in monkey superior colliculus. J. Neurophysiol..

[25] Carey D.P. (2001). Do action systems resist visual illusions?. Trends Cogn. Sci..

[26] Westwood D.A., Chapman C.D., Roy E.A. (2000). Pantomimed actions may be controlled by the ventral visual stream. Exp. Brain Res..

[27] Massendari D., Lisi M., Collins T., Cavanagh P. (2018). Memory-guided saccades show effect of a perceptual illusion whereas visually guided saccades do not. J. Neurophysiol..

[28] Tai Y.C., Lin C.H. (2019). An overview of pain in Parkinson’s disease. Clin. Park. Relat. Disord..

[29] Alexander G.E., Crutcher M.D., DeLong M.R. (1990). Basal ganglia-thalamocortical circuits: Parallel substrates for motor, oculomotor, “prefrontal” and “limbic” functions. Prog. Brain Res..

[30] Giladi N. (1997). The Mechanism of Action of Botulinum Toxin Type A in Focal Dystonia Is Most Probably through Its Dual Effect on Efferent (Motor) and Afferent Pathways at the Injected Site. J. Neurol. Sci..

[31] Scott R.B., Gregory R., Wilson J., Banks S., Turner A., Parkin S., Giladi N., Joint C., Aziz T. (2003). Executive cognitive deficits in primary dystonia. Mov. Disord..

[32] Camargo C.H., Teive H.A., Becker N., Munhoz R.P., Werneck L.C. (2011). Botulinum toxin type A and cervical dystonia: A seven-year follow-up. Arq. Neuropsiquiatr..

[33] Hok P., Veverka T., Hluštík P., Nevrlý M., Kaňovský P. (2021). The Central Effects of Botulinum Toxin in Dystonia and Spasticity. Toxins.

[34] Costanzo M., Belvisi D., Berardelli I., Maraone A., Baione V., Ferrazzano G., Cutrona C., Leodori G., Pasquini M., Conte A. (2021). Effect of Botulinum Toxin on Non-Motor Symptoms in Cervical Dystonia. Toxins.

[35] Rosales R.L., Dressler D. (2010). On Muscle Spindles, Dystonia and Botulinum Toxin. Eur. J. Neurol. Off. J. Eur. Fed. Neurol. Soc..

[36] Currà A., Trompetto C., Abbruzzese G., Berardelli A. (2004). Central Effects of Botulinum Toxin Type A: Evidence and Supposition. Mov. Disord..

[37] Sugar D., Patel R., Comella C., González D.A., Gray G., Stebbins G.T., Mahajan A. (2023). The effect of botulinum toxin on anxiety in cervical dystonia: A prospective, observational study. Park. Relat. Disord..

[38] Moriarty A., Rafee S., Ndukwe I., O’Riordan S., Hutchinson M. (2022). Longitudinal Follow-Up of Mood in Cervical Dystonia and Influence on Age at Onset. Mov. Disord. Clin. Pract..

[39] Kumar R., Dhaliwal H.P., Kukreja R.V., Singh B.R. (2016). The Botulinum Toxin as a Therapeutic Agent: Molecular Structure and Mechanism of Action in Motor and Sensory Systems. Semin. Neurol..

[40] Luvisetto S. (2021). Botulinum Neurotoxins in Central Nervous System: An Overview from Animal Models to Human Therapy. Toxins.

[41] Weise D., Weise C.M., Naumann M. (2019). Central Effects of Botulinum Neurotoxin-Evidence from Human Studies. Toxins.

[42] Mazzocchio R., Caleo M. (2015). More than at the neuromuscular synapse: Actions of botulinum neurotoxin A in the central nervous system. Neuroscientist.

[43] Restani L., Giribaldi F., Manich M., Bercsenyi K., Menendez G., Rossetto O., Caleo M., Schiavo G. (2012). Botulinum neurotoxins A and E undergo retrograde axonal transport in primary motor neurons. PLoS Pathog..

[44] Antonucci F., Rossi C., Gianfranceschi L., Rossetto O., Caleo M. (2008). Long-distance retrograde effects of botulinum neurotoxin A. J. Neurosci. Off. J. Soc. Neurosci..

[45] Aymard C., Giboin L.-S., Lackmy-Vallée A., Marchand-Pauvert V. (2013). Spinal plasticity in stroke patients after botulinum neurotoxin A injection in ankle plantar flexors. Physiol. Rep..

[46] Marchand-Pauvert V., Aymard C., Giboin L.-S., Dominici F., Rossi A., Mazzocchio R. (2013). Beyond muscular effects: Depression of spinal recurrent inhibition after botulinum neurotoxin A. J. Physiol..

[47] Opavský R., Hluštík P., Otruba P., Kaňovský P. (2011). Sensorimotor network in cervical dystonia and the effect of botulinum toxin treatment: A functional MRI study. J. Neurol. Sci..

[48] Delnooz C.C., Pasman J.W., Beckmann C.F., van de Warrenburg B.P. (2013). Task-free functional MRI in cervical dystonia reveals multi-network changes that partially normalize with botulinum toxin. PLoS ONE.

[49] Brodoehl S., Wagner F., Prell T., Klingner C., Witte O.W., Gunther A. (2019). Cause or effect: Altered brain network activity in cervical dystonia is partially normalized by botulinum toxin treatment. Neuroimage Clin..

[50] Nevrlý M., Hluštík P., Hok P., Otruba P., Tüdös Z., Kaňovský P. (2018). Changes in sensorimotor network activation after botulinum toxin type A injections in patients with cervical dystonia: A functional MRI study. Exp. Brain. Res..

